# PHEDRE trial protocol – observational study of the prevalence of problematic use of Equimolar Mixture of Oxygen and Nitrous Oxide (EMONO) and analgesics in the French sickle-cell disease population

**DOI:** 10.1186/s12888-015-0677-5

**Published:** 2015-11-14

**Authors:** Marie Gérardin, Marie-Laure Couec, Marie Grall-Bronnec, Fanny Feuillet, Laura Wainstein, Morgane Rousselet, Marie-Lyne Pinot, Fanny Perrouin, Olivier Bonnot, Marie-Hélène Drouineau, Pascale Jolliet, Caroline Victorri-Vigneau

**Affiliations:** Clinical Pharmacology Department, Nantes University Hospital, Institut de Biologie, 9 quai Moncousu, 44093 Nantes Cedex 1, France; EA 4275 SPHERE « bioStatistics, Pharmacoepidemiology and Human sciEnces REsearch, Faculties of Medicine and Pharmaceutical Sciences, Nantes University, Nantes, France; Paediatric Haematology Department, Nantes University Hospital, 7 quai Moncousu, 44093 Nantes Cedex 1, France; Addictology and Psychiatry Department, Nantes University Hospital, 85 rue de Saint-Jacques, 44093 Nantes Cedex 1, France; Biometry Platform, Nantes University Hospital, Nantes, France; Child and Adolescent Psychiatry Department, Nantes University Hospital, 7 quai Moncousu, 44093 Nantes Cedex 1, France; Paediatric Oncology and Haematology Department, Nantes University Hospital, 7 quai Moncousu, 44093 Nantes Cedex 1, France

**Keywords:** Sickle-cell disease, Equimolar Mixture of Oxygen and Nitrous Oxide (EMONO), Analgesic drugs, Problematic use, Pseudo-addiction, Substance use disorders

## Abstract

**Background:**

The use of analgesics can lead to cases of drug abuse and dependence. It can also cause pseudo-addiction in patients suffering from pain. What is the actual situation in patients suffering from severe sickle-cell disease, exposed to acute pain during vaso-occlusive crises? Evaluation of the use of analgesics, on the basis of Diagnostic and Statistical Manual of Mental Disorders criteria for substance abuse and dependence, makes it possible to differentiate the symptoms occurring only in a context of pain, in the aim of managing the pain, and thus describing pseudo-addiction, from symptoms also occurring when there is no pain, and more in favour of true addiction. Currently there is no data available in France on this problem, and no studies have been carried out in children or adolescents with sickle-cell disease. The purpose of the study is to evaluate the prevalence of problematic use of equimolar mixture of oxygen and nitrous oxide and other analgesic drugs in a population of subjects with severe sickle-cell disease in France.

**Methods/design:**

PHEDRE (Pharmacodépendance Et DREpanocytose-drug dependence and sickle-cell disease) is an observational, descriptive and transversal study. Patients under the age of 26 with sickle-cell disease are included in the study by the doctors looking after them in sickle-cell disease centres. The patients are then contacted by a trained researcher for a telephone interview, including an evaluation of the Diagnostic and Statistical Manual of Mental Disorders criteria for abuse and dependence to equimolar mixture of oxygen and nitrous oxide and for each of the analgesic drugs taken by the patient. The data are also completed using the subject’s medical record.

**Discussion:**

This study will make it possible to provide an initial quantitative and qualitative evaluation of problematic use of equimolar mixture of oxygen and nitrous oxide and analgesic drugs in the sickle-cell disease population. The results will be used firstly to provide additional data essential for monitoring the risk of overdose, abuse, dependence and misuse of these products, and to begin awareness-raising and to provide information for health care professionals, in order to significantly improve the management of sickle-cell disease-related pain.

**Trial registration:**

Clinical Trials.gov ID: NCT02580565 registered 16 October 2015

Unique Protocol ID: RC14_0344

## Background

Equimolar Mixture of Oxygen and Nitrous Oxide (EMONO) is a gas mixture that contains equal parts of each. It has Marketing Authorization (MA) in France since 2001. It is especially indicated in short-term analgesia for painful procedures or in the event of mild to moderate pain in adults and children over the age of one month.

Dependence on and tolerance to the effects of nitrous oxide have been demonstrated in animals and in humans [1–4]. Numerous cases of nitrous oxide abuse having led to adverse effects have been reported in literature [5–18]. Variability in the effects experienced with nitrous oxide is described in numerous studies [19], ranging from dysphoria to euphoria. Concerning EMONO, there is little literature on the potential of abuse, as few countries have the mixture as a drug. In the United States, the gas is delivered by pharmaceutical companies to professionals, who then perform titration themselves. Cases of severe abuse and dependence to EMONO have however been declared to the French addiction monitoring system via the network of Centres for the Evaluation of and Information on drug Dependence (Centres d’Evaluation et d’Information sur la Pharmacodépendance - CEIP-A). Analysis of these cases showed that it concerned patients in a serious medical situation, requiring repeated use of EMONO in hospital, and that a certain number of these subjects suffered from severe Sickle-Cell Disease (SCD). It therefore appeared that the SCD population could represent a high risk population for EMONO abuse or dependence. Thus, a specific data collection in these subjects was needed.

Sickle-cell anaemia and severe SCD are haemoglobinopathies from the group of corpuscular hereditary haemolytic anaemias. In France, patients with SCD are managed via a patient-oriented care network, especially Reference Centres or Special Centres for Children and Adults (RSCCA). These centres are scientific and clinical excellence structures for rare diseases such as SCD. The RSCCA medical practitioners see each patient, regardless of their age, at an inpatient/or outpatient consultation, at least once per year as part of routine follow-up. The acute pain caused by Vaso-Occlusive Crises (VOC) are the most common clinical symptom and the first cause that lead to the emergency department for children, adolescents and adults with SCD. Patients are also prone to chronic or recurrent acute pain, that require analgesics taken at the patient’s home. This pain is subject to significant inter-individual variability but also intra-individual variability, as much in terms of severity, site and duration of the pain episode [20, 21]. The unforeseeable nature of the pain and extent of the active pain relief strategies (called coping strategies), developed by patients with SCD, are different from other painful disorders in which similar analgesic treatment is used, and imply the difficulties patients and health care professionals experience in managing it [21, 22]. According to the intensity of the pain and type of treatment and according to the World Health Organisation’s (WHO) classification, level I analgesics, (acetaminophen and Non-Steroidal Anti-Inflammatory drugs (NSAIDs)), level II (codeine, tramadol, nalbuphine) and level III (morphine) can be used. EMONO is ideal for the rapid management of pain and/or in combination with morphine in hospital in children and adolescents [21, 23]. One of the barriers to management of pain in patients with SCD is the reticence of health care professionals to use morphine-based analgesics, for fear of the patient developing drug dependence [21]. Indeed, the request for analgesics can be perceived, not as a response to the pain episode but as a drug addiction [24, 25], whereas most often it is a simple clinical impression [26]. Nevertheless, undertreated pain exposes patients to the risk of “pseudo-addiction”, which occurs when patients attempt to obtain a stronger analgesic dose, thus possibly coming across resistance from the nursing team. These behaviours, which look like the compulsive search for analgesic drugs, are interpreted as being addictive-like-behaviour and widely keep the beliefs of the healthcare team as to the dependence of their patients. However in pseudo-addiction, analgesic-seeking behaviour stops when the pain is treated effectively and does not persist after pain relief has been achieved [27]. Evaluation of the use of analgesic drugs, on the basis of Diagnostic and Statistical Manual of Mental Disorders (DSM) criteria for substance abuse and dependence [28], makes it possible to differentiate the symptoms occurring only in a context of pain, in the aim of managing the pain, and thus describing pseudo-addiction (common), from symptoms also occurring when there is no/mild pain, and more in favour of true addiction (rarer). The major risk is that these patients who, in order to deal with their pain, demonstrate behaviour compatible with substance use disorder criteria, receive fewer analgesics and in whom therefore treatment is not optimal [29–31]. Also, if genuine drug addiction to analgesics was identified, it would require treatment adjustment. A specific evaluation is therefore necessary in order to improve patients’ management. The role of EMONO and opiate analgesics in the treatment strategy, the potential for abuse and dependence to which is well known, today require special monitoring of the risk of overconsumption, abuse and dependence in this population.

Currently there are no data available in France on this concern, and no studies have been carried out in children or adolescents with SCD. This lack of data is all the more significant when we know that childhood experiences and those in adolescence can determine emotions and behaviours and strategies for adaptation to events in adulthood. The main objective of this study is to evaluate the prevalence of problematic use of EMONO in a population of subjects with severe SCD treated in RSCCA in France. The secondary objectives are:

- To evaluate the prevalence of problematic use of other WHO level II and III analgesic drugs used in the treatment of pain in patients with SCD treated in RSCCA in France.
- To describe and to characterize problematic use of EMONO and other analgesic drugs.
- To study the relationships between the existence of problematic use of analgesic drugs (EMONO and/or other analgesics) and the other study evaluation parameters (sociodemographic, clinical and therapeutic data and consumption of other psychoactive substances).

## Methods/Design

### Study design

It is an observational, descriptive, transversal study aiming to determine the prevalence of problematic use of EMONO and other analgesic drugs in subjects with SCD treated in RSCCA. Subjects with SCD are recruited by medical practitioners of RSCCA. Thereafter, data are collected by a scheduled telephone interview and with subject’s medical record.

This project received the financial backing from the French National Agency for Medicines and Health Products Safety (Agence Nationale de Sécurité du Médicament et des produits de santé – ANSM) as part of the 2014 ‘Young Scientist’ request for proposals.

### Setting of the study

The CEIP-A in Nantes, is the coordinating investigation centre in charge of the general management, monitoring and coordination of the project. A consortium was set up with the partner CEIP-A in charge of coordinating the project at local level in their research area (CEIP-A in Clermont-Ferrand, Lille, Marseille, Paris and Toulouse). The RSCCA participating in the study are spread over metropolitan France territory. The RSCCA medical practitioners are in contact with the CEIP-A in Nantes and with the CEIP-A in their area. The period of inclusion for each participating medical practitioner is 12 months and the period of data collection is 15 months. There is no follow up after the patient’s assessment.

For this national multicentric study, a multi-disciplinary steering committee, comprising pharmacologists from the participating CEIP-A, medical practitioners working in RSCCA, a methodologist biostatistician, a clinical research staff and a psychiatrist specialising in addiction was set up in order to draft the study protocol. Its missions are to ensure the study is valid in terms of scientific content and methodology. The steering committee should handle any practical problems occurring during the study and where appropriate, decide on amendments to the protocol. It shall meet on a regular basis and whenever necessary, to approve aspects concerning practical organisation and to answer any questions raised during the study.

### Participants

#### Eligibility criteria

The patients are recruited for the study by their doctor when they come to the RSCCA for their routine treatment or follow-up, according to the following inclusion criteria:

- Subject with confirmed SCD diagnosis, regardless whether it is the homozygous or heterozygous form
- Subject treated for SCD at a RSCCA participating in the study, regardless of the duration of the illness
- Subject under the age of 26
- Written consent from adult subjects and written consent from one of the parents or legal guardians of minors (under 18)

The study exclusion criteria are the following:

- State-protected adult (under guardianship)
- Subject not having the general aptitude to participate in the study assessment (i.e. not able to respond to the telephone interview): too young, insufficient motor development, major difficulties understanding the French language and/or speech and/or hearing disorders.

### Patient recruitment

Each medical practitioner in the RSCCA participating in the study recruits SCD patients over a 12-month period. If the patient meets all inclusion criteria, the medical practitioner asks him, and his parents or legal guardian if the subject is a minor, if he wishes to participate in the study, and provides clear and appropriate verbal information on the study (objectives, scientific interest, and practical procedures).

- After a cooling-off period, he collects the patient’s written consent (and from at least one of the parents or the legal guardian for minors).

To facilitate schedule of the telephone interview and the collection of study data during the telephone interview, the patient has to complete two additional forms:

- The “inclusion form” is completed directly with the medical practitioner during the outpatient/inpatient consultation. This form contains identification of the RSCCA, name of the medical practitioner, name of the patient, phone number, birthdate, moment when patient can be called (days, hours). The inclusion form and written consent will be sent at the same time by secure messaging to the Nantes CEIP-A by the medical practitioner.
- The “analgesic list form” is completed by the patient alone or/and with his family (for minors) after the medical consultation but before the telephone interview. The patient should complete the form with all analgesic drugs he has taken (at home and in hospital) over the last 12 months (names and frequencies).
- Then, the medical practitioner has to complete a short clinical assessment.

The inclusion form and written consent are triplicate documents. One copy is given to the patient, one is sent by the medical practitioner to the Nantes CEIP-A, and one is kept in the RSCCA. The clinical assessment completed by the medical practitioner is also kept in the RSCCA. Once included in the study, patients will undergo a single assessment by phone, without subsequent follow-up.

### Consent for participation

At inclusion, the patients receive an information leaflet for their age range (as do the parents of minors). Then, they sign a consent form. For minors, parents also sign a consent form authorizing their children’s participation.

### Ethics statement

This non-interventional study received authorisation from the French data protection body (Commission Nationale de l’Informatique et des Libertés – CNIL) and a favourable opinion from the French advisory board for data processing in health care (Comité Consultatif sur le Traitement de l’Informatique en matière de Recherche dans le domaine de la Santé – CCTIRS) and from the local ethics committee (Groupe Nantais d’Ethique dans le Domaine de la Santé – GNEDS).

### Variables and data measurement

Data for the analysis are collected in two steps: through the clinical assessment completed by the medical practitioner and through a single telephone interview.

### Clinical assessment

After he includes a patient, the medical practitioner completes a clinical assessment which contains all information about patient’s family and personal history, history of the SCD and treatment already received for SCD. Every six months, the trained researcher will schedule a visit to the RSCCA to retrieve the clinical assessments of all patients included in this RSCCA and enter the data in the electronic case report form (e-CRF).

### Telephone interview

When Nantes CEIP-A receipts the inclusion form and written consent, the trained researcher schedules a telephone interview with the patient. Telephone interview is carried out with a trained researcher from Nantes CEIP-A. The interview is suited to the child’s, adolescent’s or young adult’s age, maturity and comprehension skills. During the telephone interview, data are stored in the electronic case report form (e-CRF). The following parameters are evaluated during the telephone semi-structured interview for EMONO, and for other analgesic drugs, during pain episodes and outside pain episodes:

1. *Prevalence of problematic use*:

Problematic use is abuse of or dependence on a substance according to the fourth edition of DSM (DSM-IV), and substance use disorder according to the fifth edition (DSM 5). At the time the protocol was written, we used DSM-IV criteria because the DSM 5 was not yet translated in French. The DSM-IV identifies seven substance dependence diagnostic criteria and four substance abuse diagnostic criteria. Abuse is defined by at least one of the four DSM-IV criteria of substance abuse and drug dependence by at least three of the seven DSM-IV criteria of substance dependence.

2. *Description and characterisation of problematic use:*

Consumption will be evaluated on the basis of quantitative and qualitative responses on the effects sought, quantitative and qualitative responses to each substance abuse and dependence criterion, by separating the pain-related criteria and non-pain-related criteria and quantitative and qualitative responses on craving.

3. *Study of the relationships between problematic use and the other study evaluation parameters*

The outcome measures are sociodemographic, clinical and therapeutic data and data on consumption of other psychoactive substances, and their relationship with problematic use of analgesic drugs. This study will be used to demonstrate any factors relating to problematic use of analgesic drugs, or even a specific analgesic drug.

### Bias

The main potential bias in this study is the heterogeneity bias during data collection. It has been prevented by the choice of a single evaluator.

### Study size

This study has never been driven among children and teenagers with SCD, which is a rare disease.

For this reason, and considering the low number of patients with SCD in France, we planned to include all those who meet the inclusion criteria in RSCCA that agreed to participate in the study. Then, we did not calculate a sample size. Before the beginning of the study, the partner CEIP-A were asked to assess the number of patients fulfilling the inclusion criteria, at their local level, in the RSCCA that agreed to participate. The total number of patients who could potentially be included in France was thus estimated at around 2000 people.

As the RSCCA medical practitioners see each patient at least once per year as part of routine follow-up, the period of inclusion was set at 12 months, to ensure that the study would be proposed to all eligible patients.

### Statistical methods

All evaluation parameters will be subjected to a descriptive analysis. The quantitative variable evaluation parameters will be described using position parameters (mean or median) and dispersion (standard deviation, interquartile range, range). Normality of their distribution will be assessed (normality test and evaluation using the relevant graphic tools). The qualitative variable evaluation parameters will be shown in the form of number and frequency tables for each modality. The primary outcome measure will be evaluated by the frequency of subjects with EMONO problematic use compared to the overall study population. The secondary outcome measures will be assessed by the frequency of subjects demonstrating problematic use of one or several other analgesic drugs, compared to the overall study population, by estimating the occurrences of positive responses for the various effects sought when taking analgesic drugs, for each DSM-IV substance abuse and dependence criterion, for each pain-related criterion, and non pain-related criterion (if the DSM-IV substance abuse and dependence criteria are met), and for craving. The frequencies will be estimated for EMONO and for each of the other analgesic drugs, the use of which is identified as problematic. The relationships between problematic use of analgesic drugs (EMONO and/or others) and the other study evaluation parameters will be assessed by univariate analysis and the appropriate statistics tests, depending on the type of variable and the size of the study population (parametric or non-parametric tests). The existence or absence of problematic use can then be studied as a dependent variable in a multivariate analysis (i.e. logistic regression) in order to test the relationship with independent variables selected from the previous analysis. No intermediate analysis or subgroup analysis is to be carried out in this study*.*

## Discussion

Recruiting patients with SCD, which is a rare disease, is a challenge for the partners working on the project. The selection criteria further restrict the number of eligible patients. Due to feasibility concerns, the entire active file of children, adolescents and young adults with SCD meeting the inclusion criteria at each participating RSCCA will be included over a 12-month period.

The purpose of the study is to evaluate problematic use of analgesic drugs, received and/or taken at the hospital and at home. Such consumption is not always noticed or identified by the subject’s friends, family or doctor. It is therefore essential to question subjects directly about this concern, without the opinion of the prescribing physician or family, and without interfering in the doctor-patient relationship. This evaluation takes place during a semi-structured interview with the subject. The inclusion criteria will have first made it possible to select only those subjects able to respond to the assessment. Given the variability in the study population, the interview will nevertheless be adapted to the child’s, adolescent’s or young adult’s age, maturity and comprehension skills. If necessary, for minors, and in order to only collect certain sociodemographic or clinical data or data relating to medicinal product consumption (not on the problematic aspect of consumption), the subject’s parent or legal guardian may also be questioned in addition to the subject.

For this multicentric study, centralised data collection by a single evaluator is necessary in order to prevent heterogeneity bias. Data collection during a scheduled telephone interview is used, on the one hand, to protect the confidentiality of the subject’s data, and on the other hand, to ensure the quality of the data collected as the subjects selected will be available. This type of consumption data (cigarettes, medicinal products, drugs etc.) collection by telephone has already been seen in a number of national surveys, such as the Baromètre Santé by the French national institute for health prevention and education (Institut National de Prévention et d’Education pour la Santé - INPES), in which subjects were compliant and the results were of good quality.

The Nantes CEIP-A is competent in the assessment of drug dependence and has recognised expertise in this field. It has conducted several studies evaluating consumption of psychoactive substances. This positive experience confirms the feasibility of these assessment methods for the Nantes CEIP-A and will therefore ensure the collection of quality data in the PHEDRE project.

The use of DSM-IV criteria could be seen as a limitation since the DSM 5 [32] is now translated and therefore usable in French. But we initially choose to assess both DSM-IV dependence and abuse criteria in addition with craving and finally, this set of criteria is consistent with the DSM 5 substance use disorders diagnostic criteria.

PHEDRE is the first study which proposes to evaluate problematic use of EMONO and analgesics in the French SDC population. We believe that differentiating pseudo-addiction from real substance use disorder will enable clinicians to adapt patient management: increase analgesia in pseudo-addiction, provide an addictological follow-up for the others.

## Abbreviations

ANSM: Agence Nationale de Sécurité du Médicament et des produits de santé (French health products agency)
CCTIRS: Comité Consultatif sur le Traitement de l’Informatique en Matière de Recherche dans le Domaine de la Santé (French advisory board for data processing in healthcare)
CEIP-A: Centre d’Evaluation et d’Information sur la Pharmacodépendance (Centre for the evaluation of and information on drug dependence and addiction monitoring)
CNIL: Commission Nationale de l’Informatique et des Libertés (French data protection body)
DSM IV: Diagnostic and Statistical Manual of Mental Disorders (4^th^ revision)
DSM 5: Diagnostic and Statistical Manual of Mental Disorders (5^th^ revision)
e-CRF: Electronic Case Report Form
EMONO: Equimolar Mixture of Oxygene and Nitrous Oxide
GNEDS: Groupe Nantais d’Ethique dans le Domaine de la Santé (Nantes ethics committee)
INPES: Institut National de Prévention et d’Education pour la Santé (French national institute for health prevention and education)
MA: Marketing Authorization
NSAID: Non-Steroidal Anti-Inflammatory Drug
RSCCA: Reference centres or Special Centres for Children and Adults
SCD: Sickle-cell disease
VOC: Vaso-occlusive crise
WHO: World Health Organisation
